# Envarsus Versus Advagraf in De Novo Kidney Transplant Recipients: A Comparative Pharmacokinetic Study

**DOI:** 10.3390/life16020256

**Published:** 2026-02-02

**Authors:** Patricio Más-Serrano, Antonio Franco, Marcos Díaz, Elena de la Cruz, Noelia Balibrea, Isabel Gascón-Ros, Amelia Ramón-López, Javier Perez-Contreras, Ricardo Nalda-Molina

**Affiliations:** 1 Pharmacy Department, University General Hospital Dr. Balmis of Alicante, Calle Pintor Baeza 10, 03010 Alicante, Spain; marcos.diaz.glez@gmail.com (M.D.); gascon_isa@gva.es (I.G.-R.); 2 Division of Pharmacy and Pharmaceutics, Department of Engineering, School of Pharmacy, Miguel Hernández University, San Juan de Alicante, 03540 Alicante, Spain; aramon@umh.es (A.R.-L.); jnalda@umh.es (R.N.-M.); 3 Alicante Institute for Health and Biomedical Research (ISABIAL-FISABIO Foundation), 03010 Alicante, Spain; antoniofrancoesteve1960@gmail.com (A.F.); eledelacruz04@gmail.com (E.d.l.C.); nobala@gmail.com (N.B.); perez_fra@gva.es (J.P.-C.); 4 Department of Nephrology, University General Hospital Dr. Balmis of Alicante, Calle Pintor Baeza 10, 03010 Alicante, Spain

**Keywords:** pharmacokinetics, calcineurin antagonists, immunosuppression, envarsus, advagraf, biological availability

## Abstract

Background: Comparative real-world data on the pharmacokinetics of once-daily tacrolimus formulations in de novo kidney transplantation remain limited. We compared tacrolimus exposure and dosing requirements with Envarsus and Advagraf during the early post-transplant period. Methods: We conducted a prospective, observational, single-center study including adult de novo kidney transplant recipients treated with once-daily tacrolimus as either Envarsus or Advagraf. The immunosuppressive protocol was based on thymoglobulin induction, with delayed initiation of tacrolimus at an initial dose of 0.15 mg/kg/day, prednisone, and sirolimus as the third immunosuppressive agent. Trough concentrations (C0), daily dose, and dose-normalized trough exposure (C0/D) were assessed at 48 h and over 3 months (days 7, 14, 30, 60, and 90). Dose adjustments were guided by therapeutic drug monitoring and Bayesian individualization to achieve target trough ranges (6–10 ng/mL during month 1; 5–7 ng/mL thereafter). Clinical effectiveness and safety outcomes were evaluated through month 3. Results: Ninety recipients were included (Advagraf n = 43; Envarsus n = 47). At 48 h, Envarsus achieved higher trough concentrations and higher C0/D than Advagraf (C0: 10.7 vs. 7.7 ng/mL; C0/D: 1.30 vs. 0.75 (ng/mL)/mg; both *p* < 0.001). From week 1 to month 3, trough concentrations were similar between groups (week 1: 8.5 vs. 8.5 ng/mL, *p* = 0.968; month 3: 5.7 vs. 5.1 ng/mL, *p* = 0.234), but Envarsus required lower daily doses (week 1: 6.4 vs. 9.9 mg/day, *p* = 0.001; month 3: 3.2 vs. 4.1 mg/day, *p* = 0.021) and maintained higher C0/D (week 1: 1.53 vs. 1.00, *p* = 0.001; month 3: 1.94 vs. 1.57 (ng/mL)/mg, *p* = 0.012). At 48 h, infra-therapeutic troughs were less frequent with Envarsus (6.7% vs. 40.5%, *p* = 0.0001), while supra-therapeutic levels were more frequent (57.8% vs. 18.9%), and tacrolimus discontinuation due to high troughs occurred more often (23.4% vs. 7.0%, *p* = 0.032). Over 3 months, the proportion of measurements within the therapeutic range was similar (57.6% vs. 64.5%, *p* = 0.705). Efficacy and safety were similar between groups. Conclusions: In de novo kidney transplant recipients, Envarsus provides higher early tacrolimus exposure and consistently higher dose-normalized trough exposure than Advagraf, enabling lower maintenance doses while maintaining similar short-term effectiveness and safety. However, early overexposure was more frequent with Envarsus at 0.15 mg/kg/day, supporting careful early monitoring and consideration of lower starting doses.

## 1. Introduction

At present time, most immunosuppressive therapy regimens used in kidney transplants include tacrolimus, given its large capacity to prevent organ rejection [1].

This drug has a narrow therapeutic index and displays a high inter- and intra-individual variability in its pharmacokinetics, making close therapeutic drug monitoring (TDM) necessary. The strong correlation between the area under the curve (AUC) and the trough concentration (trough_conc_) allows individualization of the dose only by monitoring the trough_conc_ [2,3,4,5], which simplifies the procedure enormously [5].

Tacrolimus is available in a twice-daily immediate-release formulation (Prograf, Astellas Pharma Inc., Tokyo, Japan). The effectiveness of its formulation has been tested in multiple studies [6], but its poor water solubility, metabolism in the intestinal tract, and the activity of the P-glycoprotein pump in the enterocytes mean that Prograf has a low bioavailability (around 17%) in renal transplant recipients [7].

Besides this, it is well known that compliance with the treatment increases significantly when the number of daily doses is reduced from two to one [8]. The fact that poor drug compliance is a common cause of graft loss [9] supports an additional argument for selecting once-daily dose formulations [8,9,10,11]. Therefore, it is recommended to use, from the beginning of the treatment, prolonged-release formulations instead of immediate-release tacrolimus to achieve target blood concentrations [12].

There are currently two single-dose prolonged-release tacrolimus formulations. Advagraf (Astellas Pharma Inc., Tokyo, Japan), whose efficacy and safety are similar to Prograf and have been demonstrated in numerous non-inferiority studies [13]. More recently, Envarsus (Chiesi Farmaceutici S.p.A., Parma, Italy), a recently approved new formulation based on Meltdose Release System^®^ [14], has demonstrated an increase in the bioavailability of lipophilic drugs. Envarsus combines two desired goals, to increase drug compliance and to improve bioavailability.

Previous studies in stable renal transplant patients have compared tacrolimus pharmacokinetic profiles of the conversion from Advagraf/Prograf to Envarsus [15,16]. Nevertheless, studies comparing the effectiveness of different tacrolimus in de novo transplant in ‘real life’ are scarce [12,17,18]. Budde et al. compared both Envarsus and Prograf recipients from the time of transplantation over a one-year period. They observed that those patients who received Envarsus reached therapeutic levels earlier and received significantly lower doses of tacrolimus 1 year following transplantation with similar efficacy and safety results [17]. Another study conducted by Kamar et al. assessed the pharmacokinetics of Envarsus versus Advagraf in de novo transplant. Investigators concluded that Envarsus showed better bioavailability from day 3 post-transplant. Moreover, Envarsus also achieved 30% more bioavailability than Advagraf one month after transplant with a similar safety profile [12]. In addition, a multicenter study compared Envarsus with Advagraf or Prograf during the first 6 months following de novo kidney transplantation and showed that Envarsus achieved comparable clinical outcomes while requiring a lower daily dose [18].

The aim of this study was to compare trough_conc_, doses administered, relative bioavailability, efficacy and safety of Advagraf and Envarsus, two different once-daily prolonged-release dosage forms, during the first three months after de novo kidney transplant in real-world practice.

## 2. Patients and Methods

### 2.1. Study Design and Patients

This was a prospective observational single-center study of two cohorts (January 2015–April 2017). We included white adult recipients from deceased and living donors who were maintained with the actual immunosuppression protocol from induction through three months post-transplant. Patients were stratified in two groups according to the tacrolimus formulation used: the Advagraf group and Envarsus group. To evaluate the homogeneity of both groups, patients were propensity-scored for baseline clinical characteristics and endpoint variables.

Pharmacokinetic, demographic, and other data were collected from patient medical records and the TDM database.

Ethics approval was obtained from Institutional Review Board of University General Hospital Dr Balmis of Alicante (PI2019-117; 14 April 2020).

### 2.2. Immunosuppression Protocol

The immunosuppressive protocol at the hospital with deceased donors consisted of thymoglobulin 1 mg/kg/24 h (adjusted to T lymphocyte count in peripheral blood until recovery of renal function [19]), sirolimus (initial dose 2 mg/24 h—adjusted to target therapeutic range: 4 and 8 mg/mL), prednisone (initial dose of 1 mg/kg then 20 mg/24 h at 1 month) and initiate tacrolimus (initial dose of Advagraf and Envarsus 0.15 mg/kg/24 h) after recovery of renal function (serum creatinine <3 mg/dL). Living donor recipients received the same regimen, but sirolimus and tacrolimus were started 48 h before transplant. In both groups, when tacrolimus trough_conc_ was obtained at 48 h after initiation, individualized dose adjustment though the Bayesian approach was carried out to maintain a trough_conc_ between 6 and 10 ng/mL during the first month and between 5 and 7 ng/mL thereafter. Bayesian dose individualization was performed in NONMEM using a maximum a posteriori (MAP; empirical Bayes) approach. For Advagraf, prior information was taken from the published tacrolimus population pharmacokinetic model by Woillard et al. [20]. For Envarsus, priors were based on a population pharmacokinetic model developed in the immediate post-kidney-transplantation phase [21]. Individual posterior parameter estimates were updated using observed trough concentrations together with recorded dosing and sampling times, and the recommended dose was derived to achieve the predefined target trough concentration range. Cytomegalovirus (CMV)-seronegative recipients who received grafts from seropositive donors were treated with valganciclovir for 6 months.

CMV load was monitored from the first to the sixth month post-transplant. Treatment with valganciclovir was started if the cytomegalovirus load was more than 1000 cp/mL.

### 2.3. Endpoints and Variables

Pharmacokinetics were evaluated through the analysis of tacrolimus trough_conc_, the dose administered, and the ratio between trough concentration and daily dose (trough_conc_/dose) at days 2, 7, 14, 30, 60, and 90 after starting tacrolimus. Effectiveness was assessed by the incidence of delayed graft function, acute rejection, renal function (assessed by plasma creatinine and glomerular filtration rate calculated by CKD-EPI at set times), and graft and recipient survival up to 3 months after transplantation. We collected serious adverse events such as severe infections, urological or surgical wound complications, lymphoceles, CMV infection, and neoplasia development. Immunological risk in recipients was tested calculating the percentage of panel reactive antibodies (PRAs) and the number of human leukocyte antigen (HLA) incompatibilities.

Blood samples were collected before the morning dose and tacrolimus concentration was determined in blood by enzyme-linked immunosorbent assay (TAC-DRI Thermo(R); INDIKO PLUS(R) platform (assay range: 1.2–30 ng/mL)).

### 2.4. Statistical Analysis

For quantitative variables, the mean differences with 95% confidence intervals (95% CI) or medians and percentile 25–percentile 75 (p25–p75), depending on the type of data distribution, were used. For comparison, we used the Student *t* test for independent samples or the Mann–Whitney U test.

Categorical variables were expressed in percentages, and the Chi-squared test was used for comparison. We established an alpha value of 0.05, and SPSS Software V 24 and R were used as statistical packages.

For sensitivity analyses, to explore whether a small subgroup with unusually low C0/D values (putative rapid metabolizers) could influence the pharmacokinetic comparison, we conducted a sensitivity analysis at Time 90 days, repeating the between-group comparison after excluding values below the 15th percentile of C0/D within each treatment arm. In addition, to avoid imposing a single metabolizer cut-off across formulations, we report formulation-specific C0/D tertile boundaries at Time 90 days. Additionally, we performed exploratory subgroup analyses to assess the consistency of the formulation effect across clinically relevant recipient characteristics. Participants were stratified by age (<60 vs. ≥60 years), sex, baseline body weight (<75 vs. ≥75 kg), and donor type (living vs. deceased). Within each stratum, between-group comparisons were repeated, and treatment-by-subgroup interaction was evaluated.

## 3. Results

### 3.1. Study Population

**Figure 1:**
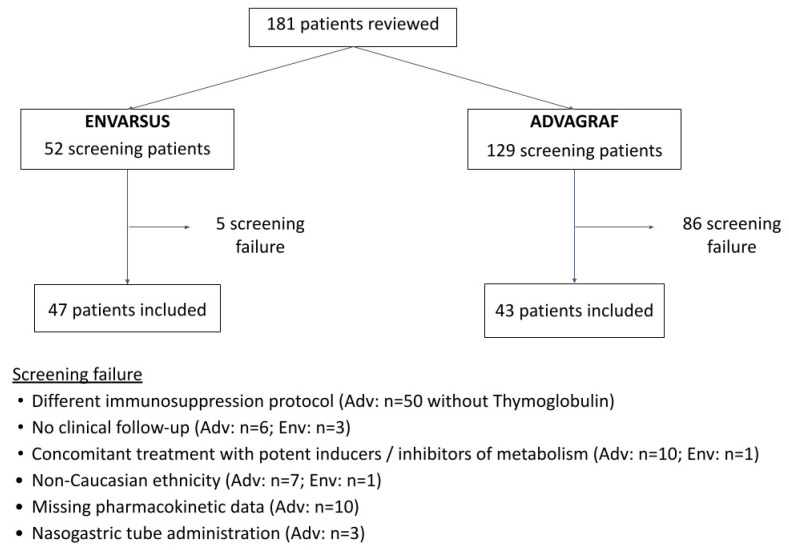
Patient flow chart.

In total, 181 renal transplant patients were identified and 90 patients were finally included in the study, 43 in the Advagraf group and 47 in the Envarsus group (Figure 1). More than half of the recipients were male (63.6%); the average age and weight were 54.2 years and 72.4 kg, respectively.

Demographic characteristics of donors and recipients (Table 1) such as age, weight, blood type, and sex, pre-transplant immune status of the recipient, pre-transplant disease status, and other transplant-related variables (living or deceased donors, percentage of re-transplants, and cold ischemia time) or those associated with treatment (trough concentration of sirolimus and steroid regimen) were similar in both groups. The median time from transplant to the introduction of tacrolimus was 5 (p25–p75: 2.0–7.8) days in patients who received Advagraf and 4 (p25–p75: 2.0–7.0) days in the Envarsus group (*p*: 0.885).

### 3.2. Pharmacokinetic Analysis

**Figure 2:**
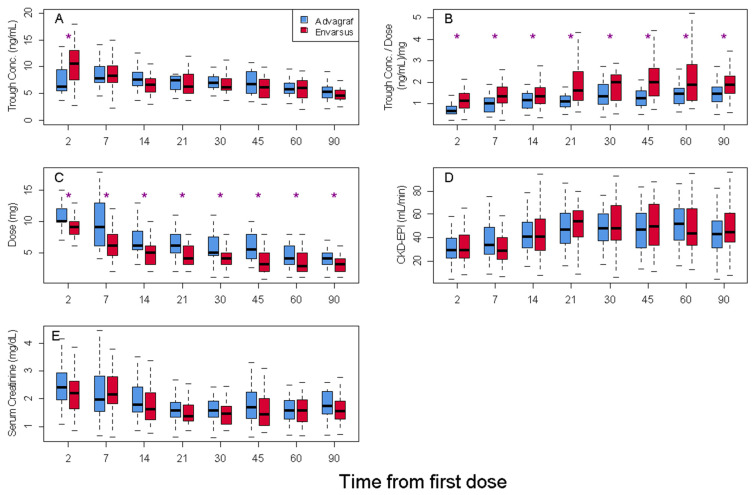
Tacrolimus Cp_trough_ boxplot (**A**), Tacrolimus Cp_trough_/dose ratio (**B**), Tacrolimus daily dose (**C**), Glomerular filtration rate (expressed as CKD-EPI) (**D**), and Serum Creatinine (**E**) for Advagraf and Envarsus groups. * *p* < 0.05.

After 48 h of tacrolimus initiation, trough_conc_ and trough_conc_/dose were significantly higher in the Envarsus group in comparison to those levels achieved in the Advagraf group (10.7 vs. 7.7 ng/mL and 1.30 vs. 0.75 (ng/mL)/mg, respectively; *p* < 0.001). However, no statistically significant differences were found in trough_conc_ in both groups from the first week until the end of the 3-month follow-up period (week 1: Envarsus: 8.5 ng/mL vs. Advagraf 8.5 ng/mL, *p*: 0.968; 3rd month: Envarsus: 5.7 ng/mL vs. Advagraf: 5.1 ng/mL; *p*: 0.234). Moreover, the administered doses of tacrolimus to maintain these trough_conc_ were significantly lower in the Envarsus group during the studied period (week 1: Envarsus 6.4 mg/day vs. Advagraf 9.9 mg/day, *p*: 0.001; third month: Envarsus: 3.2 mg/day vs. Advagraf: 4.1 mg/day, *p*: 0.021). Similarly, the trough_conc_/dose ratio was statistically superior over the whole period analyzed in the Envarsus group in comparison to the Advagraf group (week 1: 1.53 vs. 1.00 (ng/mL)/mg, *p*: 0.001; third month: Envarsus group: 1.94 vs. 1.57 (ng/mL)/mg, *p*: 0.012) (Figure 2, Supplementary Tables S1–S3). In exploratory subgroup analyses stratified by recipient age (<60 vs. ≥60 years), sex, body size (baseline body weight <75 vs. ≥75 kg), and donor type (living vs. deceased donor), Envarsus consistently exhibited a higher tacrolimus trough concentration-to-dose ratio (C0/Dose) compared with Advagraf. Formal interaction testing did not suggest clinically relevant effect modification by age, sex, body weight, or donor type (*p* for interaction = 0.139, 0.727, 0.972, and 0.440, respectively, Supplementary Table S4).

**Figure 3:**
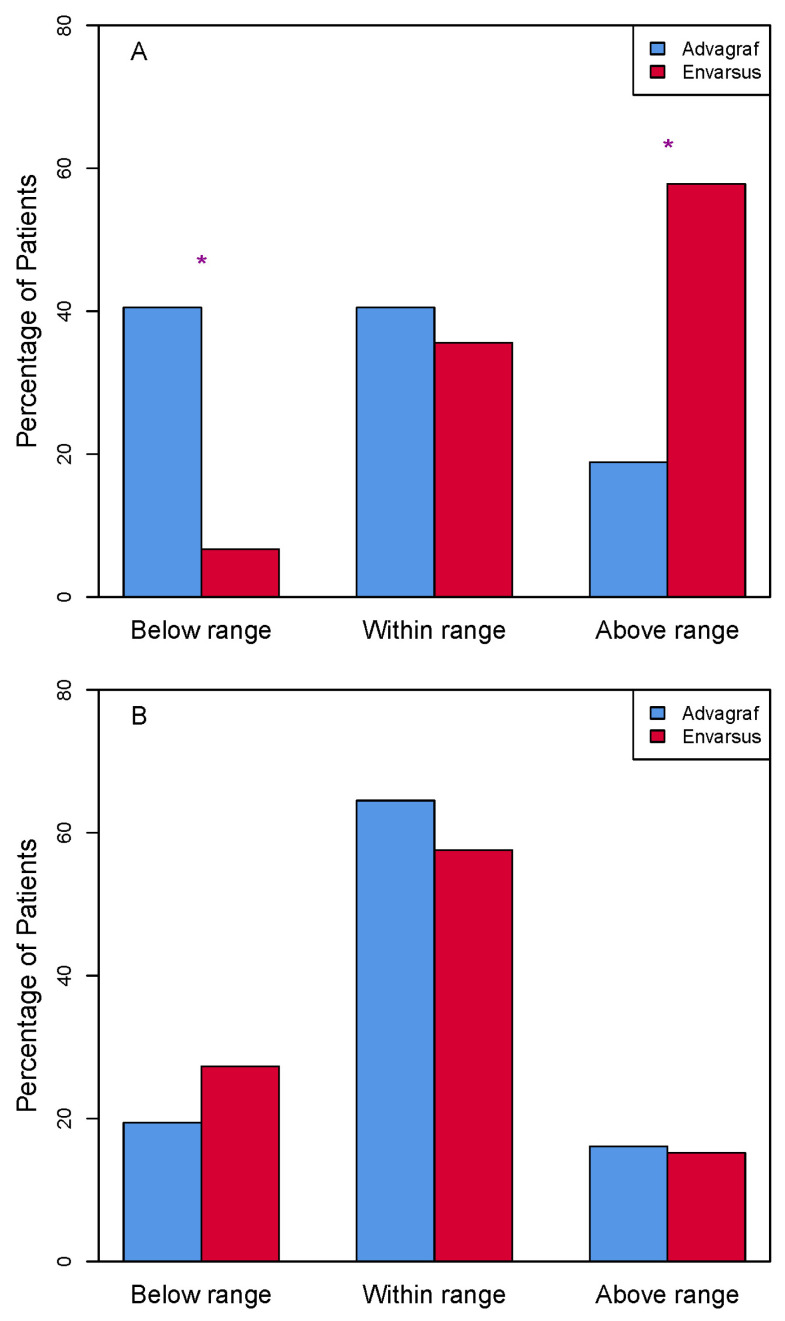
Percentage distribution of tacrolimus Cptrough in relation to the therapeutic range at (**A**) 48 h after start of tacrolimus and (**B**) during 3-month follow-up. * *p* < 0.05.

At 48 h after starting tacrolimus therapy, 6.7% and 40.5% of patients in the Envarsus and Advagraf groups, respectively, showed infra-therapeutic levels (*p*: 0.0001); however, lower percentage of patients in the Advagraf group had trough_conc_ above the upper limit of the therapeutic range (57.8% vs. 18.9% in the Envarsus and Advagraf group, respectively), while near half of the patients in each group were within the therapeutic range (Figure 3A). Also, in 23.4% and 7% of transplant patients, tacrolimus was discontinued due to high trough_conc_ in the Envarsus and Advagraf group, respectively (*p*: 0.032). During the three months of follow-up, the percentage of patients within the therapeutic range was similar in both groups (Envarsus 57.6% vs. Advagraf 64.5%, *p*: 0.705) (Figure 3B).

### 3.3. Efficacy and Safety

Efficacy and safety variables such as renal filtration (Figure 2), incidence of delayed graft function, acute rejection, acute tubular necrosis, urological or wound complications after surgery, serious infection, CMV infection, loss of graft, and deceased patients after the 3-month period were similar in both groups (Table 2).

## 4. Discussion

This cohort study carried out in our center assessed the pharmacokinetics of two different prolonged-release tacrolimus formulations in clinical practice over a 3-month post-transplant period.

We observed that 48 h after the initial dose of tacrolimus administered by protocol, trough_conc_ in the Envarsus group was significantly higher than in the Advagraf group. To achieve the same trough_conc_, the required dose of Envarsus was significantly lower than the Advagraf dose. This fact resulted in a trough_conc_/dose ratio statistically superior in the Envarsus group (23% greater at 3 months), which reflected a better relative bioavailability due to the formulation based on Meltdose Release System^®^ [14].

These results are similar to those obtained by Kamar et al. [12], where the authors used the AUC/daily dose ratio as an index to compare the relative bioavailability of both formulations (Envarsus vs. Advagraf). Although there were no differences in the relative bioavailability on the first day post-transplant, a significant difference was then observed from the 36 h until the day 28 post-transplant [12]. This initial difference in their results compared to ours could be attributed to the postsurgical changes and the alteration in the intestinal tract functioning at the immediate postoperative period [22]. In our study, treatment with tacrolimus was initiated a median of 4–5 days after surgery, avoiding this potential interaction. In addition, the first trough_conc_ of tacrolimus was analyzed after the administration of two doses. Although tacrolimus trough_conc_ is not expected yet to reach steady-state levels, it is more representative of drug exposure than the one achieved after a single-dose administration as in the study of Kamar et al. [12]. The AUC/dose ratio represents a reliable marker of relative bioavailability. However, given the complexity of measuring AUC in clinical practice at different time points, it was decided to use the trough_conc_/dose ratio, also used by other authors [23]. Our results are also similar to those reported by the European multicenter study, in which Envarsus achieved similar clinical outcomes to standard-of-care tacrolimus (Prograf or Advagraf) with a lower weight-normalized total daily dose from week 3 to month 6 after transplant [18], but they focus the comparison from week 3 to month 6 after transplant, instead of in the initial period.

According to our data, relative bioavailability of Envarsus compared to Advagraf remained constant during the three months of follow-up (Figure 2B), contrary to Budde’s study, in which an improvement in trough_conc_/dose ratio was observed during the period of study. However, in this case, the comparison group was Prograf, the patients in the Envarsus group received a higher initial dose (0.17 mg/kg vs. 0.1 mg/kg), and the follow-up period was longer [17].

The Kidney Disease Improving Global Outcomes (KDIGO) clinical practice guidelines indicate that the sooner the target levels of a calcineurin inhibitor are reached, the greater the efficacy of these drugs at preventing acute rejection [24]. Despite label recommendations in renal transplantation (Envarsus: 0.17 mg/kg/24 h [25]; Advagraf: 0.2–0.3 mg/kg/24 h [23]), the initial dose in our institutional protocols for both formulation (Envarsus: 0.15 mg/kg/24 h; Advagraf: 0.15 mg/kg/24 h) was lower based on our previous experience in the conversion study [15] and because our immunosuppressive protocol includes the use of thymoglobulin in all patients. Other authors like Krzyżowska et al. had used the same initial doses of Advagraf (0.15 mg/kg) without changes in graft renal function or acute rejection rates [26]. It should be noted that the initial doses of Envarsus used by Kamar [12] and Budde [17,18] were higher than those used in our series. Budde [17] reported that 36.6% of patients treated with Envarsus achieved levels within the therapeutic range 24 h after drug initiation, while in our study, only 6.7% of patients had infra-therapeutic levels 48 h later. Also, half of patients were within the therapeutic range in both groups, although near 40% of patients were below the target trough_conc_ in the Advagraf group and more than 50% of patients in the Envarsus group were above the therapeutic range. So, it is possible that despite having initiated the treatment with a lower dose in the Envarsus group than the label one, 0.15 mg/kg is still high, since there is a large percentage of patients in the Envarsus group that needed to discontinue the drug during 24 h because of high trough_conc_. As a matter of fact, we have recently reported in a larger prospective cohort that an even lower induction dose of Envarsus (0.08 mg/kg) is safe and provides the best balance between adequate exposure and avoidance of early overexposure [27]. The dose adjustment through the Bayesian approach allowed trough_conc_ on day 7 to become similar for both formulations, long before it did in the series by Budde et al. [17]. Taken together, these data suggest that the label-recommended initial dose of Envarsus may be higher than necessary and that lower starting doses (<0.15 mg/kg/day), in line with more recent dose-finding studies, including our own evaluation of different initial doses [12,18,25,27], are sufficient for the induction phase while reducing the risk of early overexposure.

In our study, dosage differences in the Envarsus group compared with Advagraf ranked between 35% and 22% at the first week and at the third month, respectively. These findings are consistent to what was evidenced in the study of Kamar et al., which observed a significantly lower daily dose to achieve the same trough_conc_ in the Envarsus group compared with Advagraf group with a dose reduction of 40% on day 28 post-transplant [12]. This dose reduction has also been observed in studies of conversion from Advagraf to Envarsus in stable renal transplant, with similar dose reduction percentages [15,16]. The same results have been found when comparing Envarsus with an immediate-release formulation in de novo kidney transplantation, at week 4, during the follow-up year [17] and after 2 years [28], where the dose required to achieve similar trough_conc_ was lower in the Envarsus group, at 6.9%, 18.3%, and 24%, respectively.

Regarding the follow-up period, we acknowledge that a 12-month observation would provide more robust data on long-term clinical outcomes such as chronic nephrotoxicity or graft survival. However, the primary focus of this study was to characterize the relative bioavailability and the stabilization phase in the immediate de novo period, which is the most critical window for pharmacokinetic variability and dose-related acute toxicity. Previous large-scale, randomized trials, such as those by Budde et al. and Rostaing et al. [17,18,26], have already established the long-term non-inferiority of Envarsus compared to other formulations at 12 and 24 months. Our research complements these findings by providing “real-world” insights into the first 90 days, demonstrating that the superior bioavailability of Envarsus is evident from the very first doses. This early period is essential for Model-Informed Precision Dosing (MIPD) strategies, as achieving target concentrations swiftly and safely is known to influence long-term graft success [24].

One of the limitations of this study is that the design is based on real-world data and lacks the strength of a randomized clinical trial, such as the European multicenter study by Budde et al., which also demonstrated that Envarsus can achieve similar efficacy and safety to standard-of-care tacrolimus [18]. However, the cohorts are very homogenous and close in time, and a propensity score was performed. The fact that the design of the study required the use of the same initial dose of tacrolimus in both groups eliminated a possible bias attributable to the dosage in the immediate post-transplant period. Multiple studies, like the one conducted by Stratta et al., have shown changes in trough_conc_ of tacrolimus related to the age, weight, and gender of the recipient, steroid dose received, or infection with hepatitis C virus [23]. In the present study, demographics, levels of m-TOR (sirolimus), and the percentage of patients with tacrolimus trough levels within the therapeutic range during the 3-month follow-up were also statistically similar in both groups, and none of our recipients carried the hepatitis C virus. Some studies included a significant number of black recipients [2,28,29,30]. According to Tremblay et al., this population has demonstrated significantly higher dose requirements than white populations to reach the same trough_conc_ [16]. All donors and recipients were of the same race and came from a very limited geographical territory, as in Kamar et al.’s study [12]. In addition, all recipients underwent kidney transplant at the same hospital, which unifies surgical, immunological, pharmacokinetic, and handling criteria, which resulted in consistency of results and represents the real-world data of our clinical practice. Also, we explored two of the weaknesses identified in the study of Kamar et al. [12], because all patients were treated with thymoglobulin (instead of basiliximab) and were studied for a longer period of time (3 months), a critical period for the development of acute rejection and opportunistic infections. All of this makes both groups comparable, thus avoiding potential bias associated with observational studies. We compared two arms, Envarsus against Advagraf, but the European multicenter study designed one arm, which included recipients receiving Prograf or Advagraf, with a very heterogeneous immunosuppression regimens, which could develop potential bias [18].

In conclusion, Envarsus improves tacrolimus bioavailability compared to Advagraf in de novo kidney transplantation patients, achieves trough concentration faster and higher when using an initial dose of 0.15 mg/kg of both drugs, and enables a significant reduction of near 25% of the dose without changes in renal function or patient and graft survival. Further reductions in the label initial doses of Envarsus seem to be needed based on the percentage of patients with trough concentration above the therapeutic range.

Due to tacrolimus’ narrow therapeutic index and different relative bioavailability, both formulations are not interchangeable.

## Figures and Tables

**Table 1:** Baseline characteristics of the study population.

	Advagraf	Envarsus	*p*
Patients (n)	43	47	-
Weight, mean (CI95% ^1^)	73.5 (69.1–77.2)	72.6 (66.5–74.7)	0.344
Recipient gender (M/F) ^2^, %	76.7/23.3	55.3/44.7	0.051
Donor gender (M/F), %	65.8/34.2	60.9/39.1	0.658
Recipient age, median (p25–p75) ^3^	54 (46–61)	55 (48–67)	0.287
Donor age, median (p25–p75)	51 (43–60)	53 (48–66)	0.511
Type of renal replacement therapy (RRT)			
Hemodialysis	55.6%	46.7%	0.732
Peritoneal dialysis	22.2%	26.7%	
Pre-dialysis	22.2%	26.7%	
Cold ischemia time (h), median (p25–p75)	17.5 (13–21)	20 (15–23)	0.172
Time until tacrolimus initiation (days), median (p25–p75)	5 (2–7.75)	4 (2–7)	0.883
Blood type			
A	44.4%	40%	0.498
B	5.6%	6.7%	
AB	5.6%	0%	
O	44.4%	53.3%	
CMV donor + recipient -	3/7%	4/8.5%	0.826
Living donor, n (%)	3/7%	5/10.6%	0.716
Retransplant, n (%)	10/23.3%	10/21.3%	1.000
HLA ^4^ incompatibility mismatch (%)			
4–6	53.2	51.1	0.834
0–3	46.8	48.9	
PRA ^5^ > 50%	4.7	2.1	0.465
Sirolimus trough_conc_ (day 14), median (p25–p75)	6.5 (4.6–8.6)	6.2 (4.9–7.4)	1.000

^1^ CI95%: confidence interval 95%, ^2^ (M/F): male/female; ^3^ p25–p75: percentile 25–percentile 75. ^4^ HLA: human leukocyte antigen; ^5^ PRA: virtual panel-reactive antibody. CMV: cytomegalovirus.

**Table 2:** **Efficacy and safety data.**

	Advagraf	Envarsus	*p*
Delayed renal function, n (%)	3/7%	4/8.5%	1.000
Acute rejection, n (%)	1/2.3%	3/6.4%	0.618
Urological complication, n (%)	5/11.6%	11/23.4%	0.175
Lymphocele, n (%)	3/7.0%	4/8.5%	1.000
Wound complication, n (%)	6/14%	56/12.8%	1.000
Severe infection, n (%)	4/9.3%	3/6.4%	0.705
CMV ^1^, n (%)	5/11.6%	8/17.0%	0.556
Neoplasia, n (%)	0/0%	0/0%	1.000
Graft loss, n (%)	3/7.0%	4/8.5%	1.000
Deceased patients, n (%)	2/4.7%	1/2.1%	0.604

^1^ CMV: cytomegalovirus.

## Data Availability

Data is unavailable due to privacy and ethical restrictions.

## Supplementary Materials

The following supporting information can be downloaded at: https://www.mdpi.com/article/10.3390/life16020256/s1, Figure S1: Individual tacrolimus trough concentrations over time (spaghetti plots); Table S1: C0/D distribution at Time 90 days; Table S2. Sensitivity analysis excluding <P15 within each group; Table S3. Formulation-specific tertile boundaries at Time 90 days; Table S4. Exploratory subgroup analyses of tacrolimus trough concentration-to-dose ratio (C0/Dose) at Time 90 days).

## Abbreviations

AUC	area under the blood concentration–time curve
TDM	Therapeutic Drug Monitoring
trough_conc_	trough concentration
PRA	panel-reactive antibody
HLA	human leukocyte antigen
CMV	cytomegalovirus
CI95%	confidence interval of 95
P25–P75	25th and 75th percentiles
