# Supplementary material for: Envarsus Versus Advagraf in De Novo Kidney Transplant Recipients: A Comparative Pharmacokinetic Study
Source: Life (Basel). 2026 Feb 2;16(2):256. doi: 10.3390/life16020256 (PMC12942462; doi:10.3390/life16020256)
Supplement: Supplementary file 1 [file life-16-00256-s001.zip › life-4058577-supplementary.pdf]

Supplementary Figure S1. Individual tacrolimus trough concentrations over time (spaghetti plots)

Individual patient trajectories of tacrolimus trough concentrations (C0, ng/mL) during the first 90 days after tacrolimus initiation are shown separately for Advagraf and Envarsus. Each line represents one patient and points correspond to measured trough concentrations at the scheduled visits (approximately 48 h and days 7, 14, 30, 60 and 90). Horizontal shaded bands indicate the predefined therapeutic target ranges (6–10 ng/mL during the first month and 5–7 ng/mL thereafter).

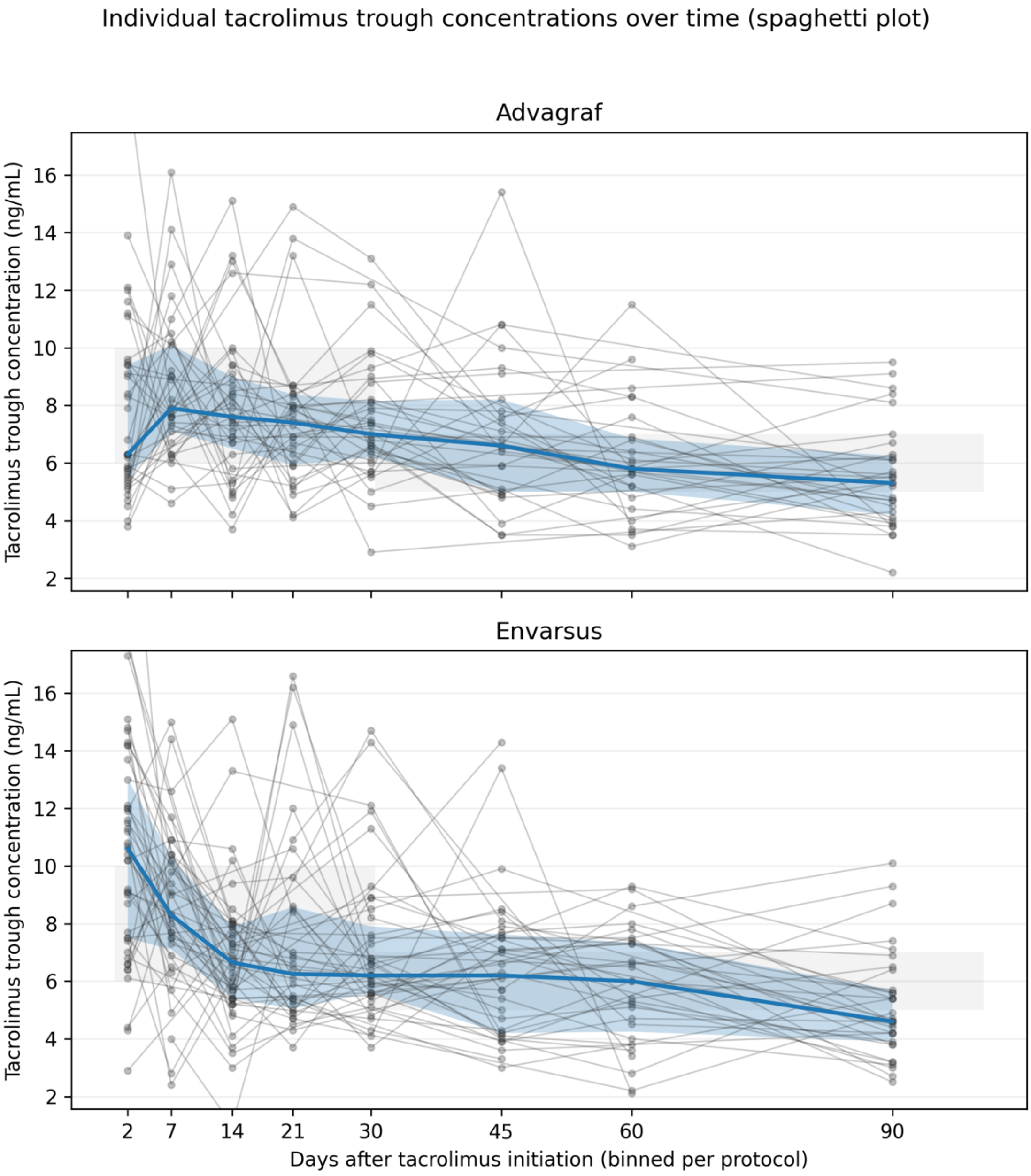

At Time 90 days, we (i) summarized the C0/D distribution using formulation-specific tertiles, and (ii) performed a robustness check excluding the lowest tail of the C0/D distribution by removing values below the 15th percentile (P15) (n=5) within each formulation and repeating the between-group comparison.

Table S1. C0/D distribution at Time 90 days

Group	Mean	Median	SD	Skewness	Excess kurtosis	P15	P33	P67
**Advagraf**	1.546	1.433	0.705	1.450	2.681	0.945	1.175	1.620
**Envarsus**	1.936	1.860	0.873	0.222	-0.623	0.829	1.622	2.178

Skewness >0 indicates right-skewness. Kurtosis is reported as **excess kurtosis** (0 corresponds to normal distribution).

Table S2. Sensitivity analysis excluding <P15 within each group

Analysis	Advagraf mean	Envarsus mean	Advagraf median	Envarsus median	Mann–Whitney p
**Baseline (all patients at time 90 days)**	1.546	1.936	1.433	1.860	0.0379
**Sensitivity (exclude <P15 within each group)**	1.693	2.160	1.496	2.050	0.00591

Table S3. Formulation-specific tertile boundaries at Time 90 days

Formulation	T1 (lowest)	T2	T3 (highest)
**Advagraf**	< 1.175	1.175–1.62	≥ 1.62
**Envarsus**	< 1.622	1.622–2.178	≥ 2.178

Table S4. Exploratory subgroup analyses of tacrolimus trough concentration-to-dose ratio (C0/Dose) at Time 90 days

Subgroup	Level	C0/Dose median (Advagraf)	C0/Dose median (Envarsus)	P (within subgroup)	P for interaction
**Age**	<60 years	1.050	1.470	0.0006	0.139
	>=60 years	1.513	1.670	0.666	
**Sex**	Male	1.135	1.642	0.015	0.727
	Female	1.095	1.520	0.030	
W**eight**	<75 kg	1.095	1.595	0.0056	0.972
	>=75 kg	1.135	1.633	0.033	
**Donor type**	Deceased donor	1.178	1.577	0.003	0.440
	Living donor	0.925	1.880	0.400	
